# Omadacycline for treatment of acute bacterial infections: a meta-analysis of phase II/III trials

**DOI:** 10.1186/s12879-023-08212-0

**Published:** 2023-04-14

**Authors:** Fei Lin, Rong He, Bin Yu, Bowen Deng, Baodong Ling, Mingyong Yuan

**Affiliations:** 1 Department of Pharmacy, The First Affiliated Hospital of Chengdu Medical College, Chengdu, China; 2 Clinical Medical College, Chengdu Medical College, Chengdu, China; 3 Department of Respiratory and Critical Care Medicine, The First Affiliated Hospital of Chengdu Medical College, Chengdu, China; 4 Department of Pharmacy, Mianyang Central Hospital, Mianyang, China; 5 Department of Pharmacy, The Sixth People’s Hospital of Chengdu, Chengdu, China; 6 School of Pharmacy, Chengdu Medical College, Chengdu, China; 7 Outpatient Department, The First Affiliated Hospital of Chengdu Medical College, Chengdu, China

**Keywords:** Omadacycline, Acute bacterial infections, Meta-analysis, Efficacy, Safety

## Abstract

**Objective:**

This study aims to assess the clinical efficacy and safety of omadacycline for the treatment of acute bacterial infections.

**Methods:**

A search of PubMed, Embase, Cochrane Library, and Clinical Trials was conducted up to July 1, 2022. We included only randomized controlled trials (RCTs), in which omadacycline and other antibiotics were evaluated for treating acute bacterial infections in adults. The primary outcomes were clinical response and microbiological response, whereas the secondary outcome was the risk of adverse events (AEs).

**Results:**

A total of seven RCTs involving 2841 patients with acute bacterial infection were included. Overall, our study illustrated that the clinical cure ratio of omadacycline was similar to the comparators in the treatment of acute bacterial infections (OR = 1.18, 95%CI = 0.96, 1.46, I^2^ = 29%). Omadacycline had a microbiological eradication rate similar to comparators in the treatment of acute bacterial infections (OR = 1.02, 95%CI = 0.81, 1.29, I^2^ = 42%). No statistical differences were observed between omadacycline and the comparators in terms of infection caused by *Staphylococcus aureus* (OR = 1.14, 95%CI = 0.80, 1.63, I^2^ = 0%), methicillin-resistant *S. aureus* (MRSA, OR = 1.28, 95%CI = 0.73, 2.24, I^2^ = 0%), methicillin-susceptible *S. aureus* (MSSA, OR = 1.12, 95%CI = 0.69, 1.81, I^2^ = 0%), and *Enterococcus faecalis* (OR = 2.47, 95%CI = 0.36, 16.97, I^2^ = 7%). A significant difference was found between omadacycline and the comparators for the risk of any AEs and treatment related AEs. The risk of discontinuation of the study drug due to an AEs was lower for omadacycline than for the comparators.

**Conclusion:**

Omadacycline is as good as comparators in terms of efficacy and tolerance in the treatment of acute bacterial infections in adult patients. Thus, omadacycline is an appropriate option for antibiotic therapy in adult patients with acute bacterial infections.

## Introduction

One of the greatest concerns of recent time is antibacterial drug resistance. It is a global issue that requires long-term action. Infections caused by drug-resistant bacteria are a growing health threat, and they are getting worse [1, 2]. Antibiotic-resistant bacteria are estimated to cause approximately 700,000 deaths worldwide each year, with over 10 million expected by 2050 [3, 4]. To treat drug-resistant bacterial infections, new antimicrobial ceftazidime/avibactam, ceftolozane/tazobactam, delafloxacin, eravacycline, omadacycline, meropenem/vaborbactam, and imipenem/relabactam, etc. have been developed [4, 5].

Omadacycline (Nuzyra), tetracycline class, third-generation aminomethylcycline antibacterial agent, was approved by the Food and Drug Administration (FDA) and European Medicines Agency (EMA) for the treatment of community acquired bacterial pneumonia (CABP) and acute bacterial skin and skin structure infections (ABSSSI) in adults [6, 7]. In addition, omadacycline is being used to treat a variety of bacterial infections, including urinary tract infections and other community-acquired infections. In comparison to tigecycline, a glycosamine-based, tetracycline-class drug for the treatment of antibiotic-resistant bacteria, omadacycline differs by only one carbonyl group [1, 2, 8]. Omadacycline is a once-daily orally or intravenously administered antibiotic that overcomes the resistance to tetracycline and has broad antimicrobial activity against clinical pathogens, including gram-positive, gram-negative, atypical pathogens and multidrug-resistant isolates [9, 10]. In vitro, omadacycline was active against both methicillin-resistant *S. aureus* (MRSA), methicillin-susceptible *S. aureus* (MSSA), vancomycin-resistant *E. faecium*, penicillin-resistant *Streptococcus pneumonia*e, extended-spectrum β-lactamase (ESBL) positive *Escherichia coli*, ESBL-negative *E. coli*, and carbapenem-resistant *Acinetobacter baumannii* with MIC_90_ values of 0.25 mg/L, 1 mg/L, ≤ 0.06 mg/L, 0.12 mg/L, 4 mg/L, and 4 mg/L, respectively [11].

Recently, some studies found that omadacycline has good clinical activity with a relatively low risk of adverse events (AEs) than other antibiotics [12, 13]. AEs defined as emerged after treatment initiation with onset or worsening of severity that occurred at or any time after administration of the first dose of trial drug through the final follow-up visit. In recent years, with the emergence of drug-resistant bacteria and the expansion of the indication of omadacycline in acute bacterial infections, it is necessary to systematically evaluate the clinical efficacy and safety of omadacycline in the treatment of acute bacterial infections. Therefore, we selected omadacycline as the research object to compare its clinical efficacy and safety in the treatment of acute bacterial infections (complicated akin and skin structure infection, ABSSSI, CABP, cystitis, and acute pyelonephritis), in order to provide real-time evidence for clinical application.

## Methods

### Data searches and study selection

The Preferred Reporting Items for Systemic Reviews and Meta-Analyses (PRISMA) statement was followed for conducting this study [14]. We carried out a systematic search on PubMed, Embase, Cochrane Library, and Clinical Trials for articles published up to July 1, 2022, to identify all study assessing omadacycline therapy for patients with acute bacterial infections, using the search terms: ‘omadacycline’ OR ‘Nuzyra’ OR ‘PTK-0796’. Studies published only in English were included. The duplicate records were removed by using EndNote X8, and two reviewers (He and Yu) examined records independently to avoid bias. If any disagreement occurred in the process, it was resolved by a third reviewer (Lin). Randomized controlled tails (RCTs) that compared the clinical efficacy response and safety of omadacycline and other antibiotics in the treatment of acute bacterial infections were included. Excluded studies included in vitro, pharmacokinetic/pharmacodynamic, those without a comparator group and not RCT.

### Data extraction and quality assessment

The outcome data was extracted independently by two researchers who used a standardized form. In the case of disagreements during data extraction, the issue was checked and resolved by the third researcher. Authorship, publication year, the design of the study, study population characteristics, intervention drug regimens, efficacy outcome (clinical response and microbiological response), and safety outcome of adverse events (AEs) were extracted from all included studies. The risk of bias of all included studies was determined and rated as “low risk”, “high risk,“ or “unclear risk” according to the items of the Cochrane Collaboration’s Risk of Bias Tool, version 2.0 [15].

### Statistical analyses

On dichotomous data, we used a random-effects model to calculate intervention effect odds ratios (ORs) with 95% confidence intervals (CIs). Cochran’s Q test and the I^2^ statistic were used to assess the proportion and degree of heterogeneity. P < 0.10 or I^2^ > 50% for the Q-test was regarded as a significant value. If I^2^ > 50%, a random-effects model was performed in the presence of high heterogeneity; otherwise, a fixed-effects model was applied. Statistical analyses were carried out with Review Manager version 5.3, and statistical significance was determined as a P-value < 0.05.

## Results

### Study identification and study characteristics

**Fig. 1:**
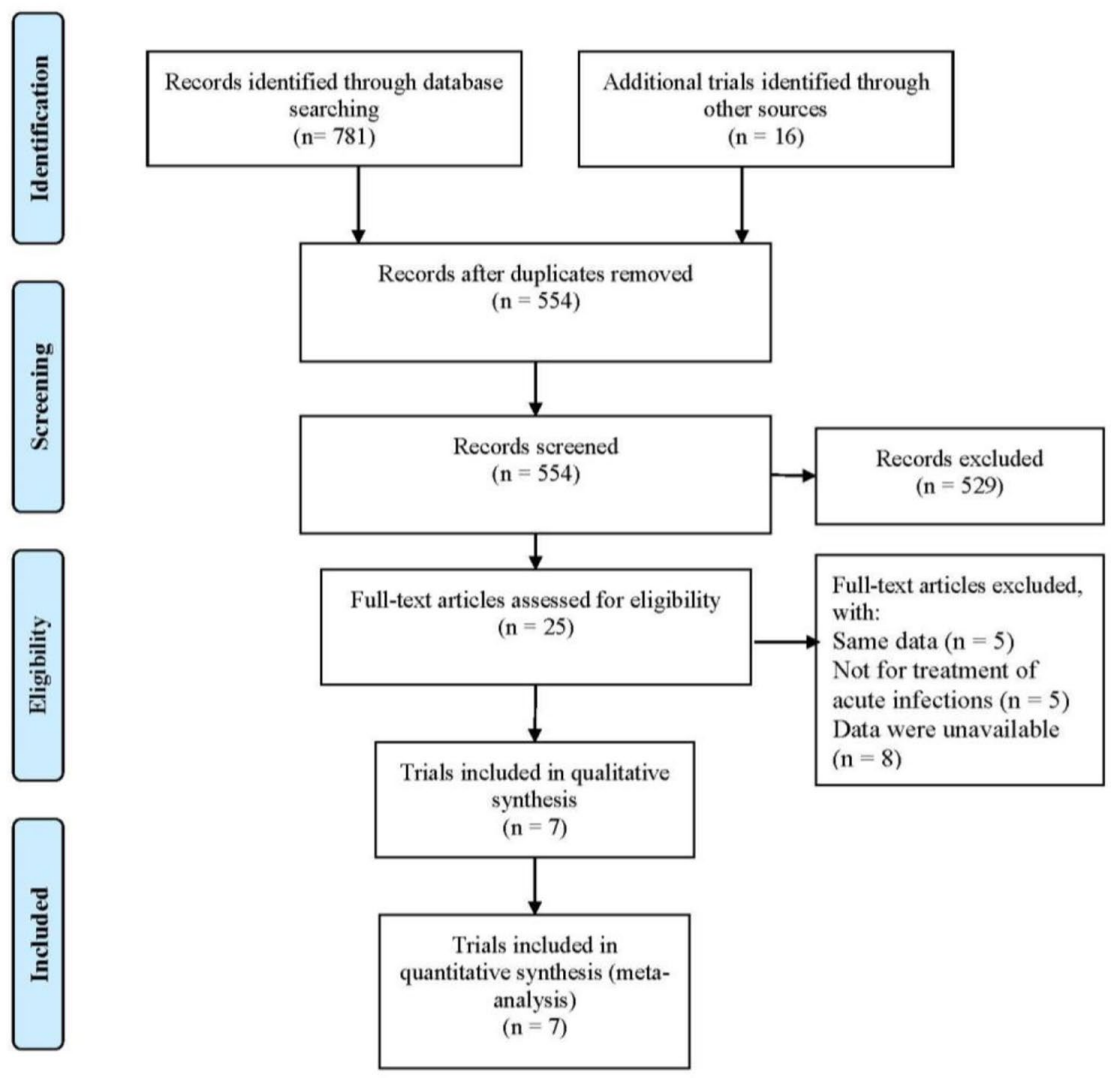
Flow diagram in this meta-analysis

A flow diagram of study selection is presented in Fig. 1. Initial database search resulted in 797 records, including PubMed (N = 220), EMBASE (N = 502), Cochrane Library (N = 59), and Clinical Trials (N = 16). After excluding 243 duplications, the remaining 554 title and abstract records were screened, with 529 records being excluded. Twenty-five records were found to be relevant for further detailed evaluation. Of these, 18 records were excluded as having the same data (N = 5), not being for the treatment of acute infections (N = 5), and having data that was unavailable (N = 8). Finally, eligible 7 RCTs [16–19] were included in this meta-analysis.

**Fig. 2:**
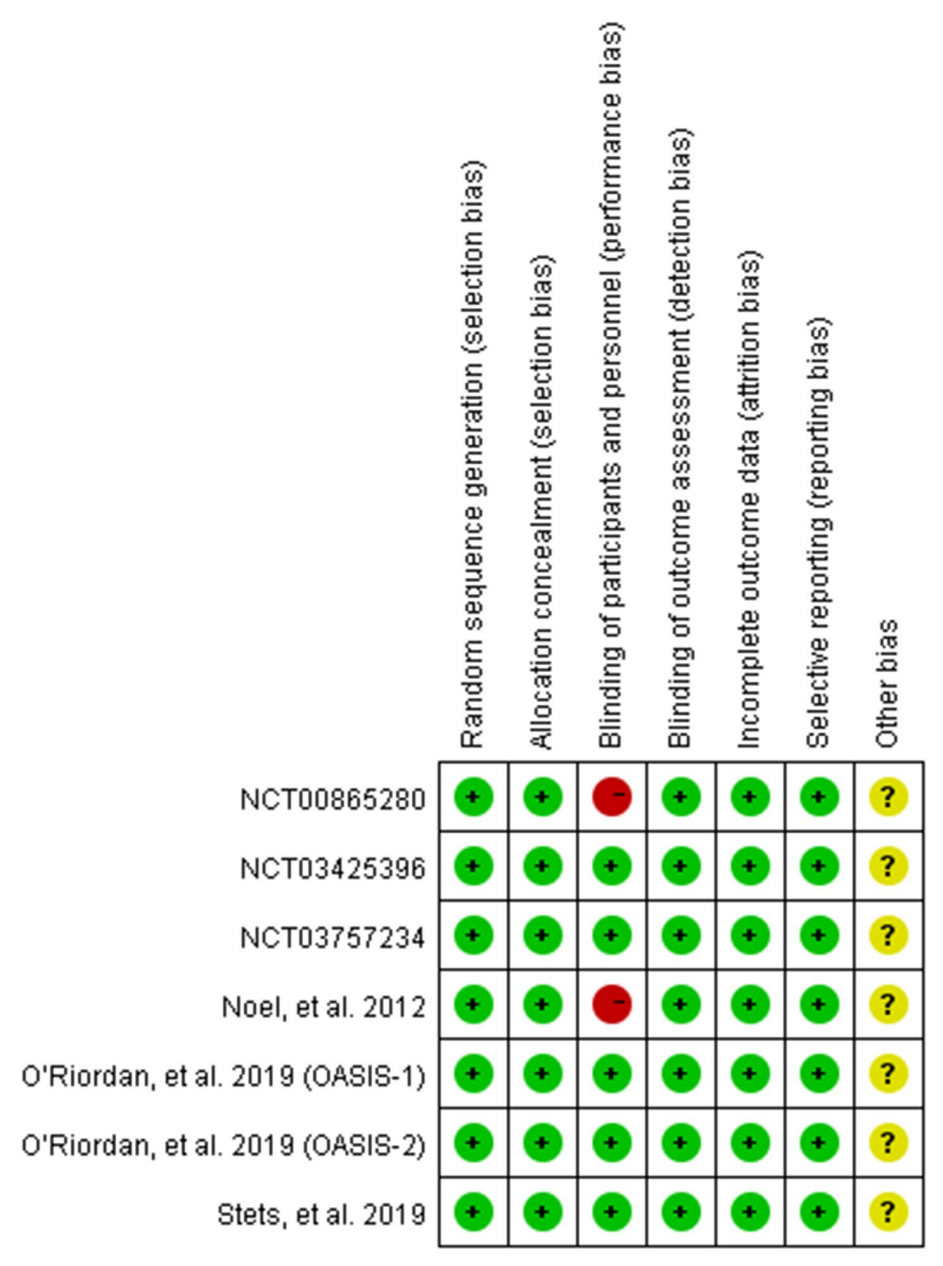
Quality assessment for risk of bias for studies +: lower risk, -: higher risk, ?: unclear risk

**Fig. 3:**
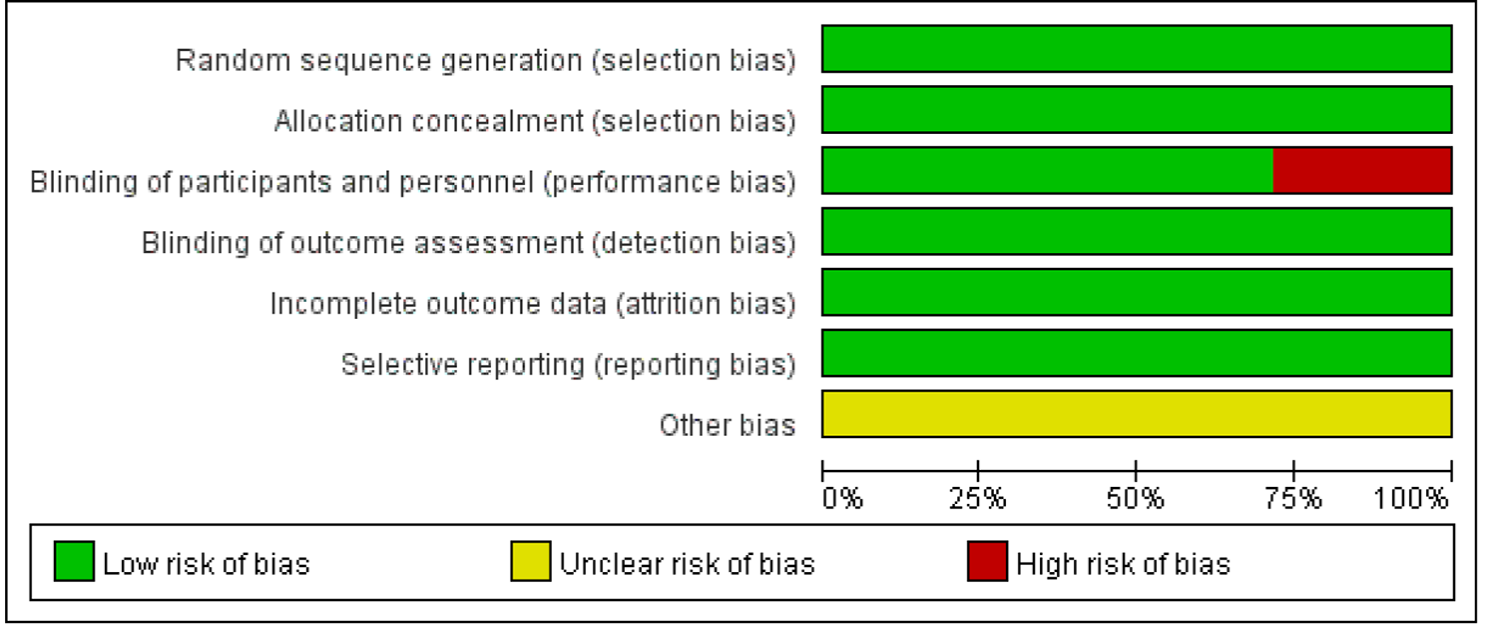
Graphs of risk of bias for studies

The characteristics of 7 RCTs are summarized in Table 1, with a total of 2841 patients were enrolled in the meta-analysis. The number of patients ranged from 54 to 388 subjects. The experimental groups that received omadacycline and other antibiotics (linezolid, moxifloxacin, nitrofurantoin, and levofloxacin) consisted of 1,563 and 1,278 patients, respectively. Of these, four studies were published in full text between 2012 and 2019 [16–19], and three additional eligible studies (NCT00865280, NCT03425396, and NCT03757234) were completed but unpublished. Five studies were double-blind, and two studies were evaluator-blind. Four studies were conducted in only the United States, and the other three studies were conducted in multiple countries. Three studies used linezolid as a comparator; one used nitrofurantoin; one used levofloxacin; one used moxifloxacin; and one used l either inezolid or moxifloxacin. The risk of bias of the included studies is presented in Figs. 2 and 3, and only two studies had a high risk of bias in the domains of blinding of participants and performance.

**Table 1:** The character and baseline of 7 RCTs

Study, year published	Study duration	Study site	Study population	No of population (n)			Dose regimen	
				Omadacycline	Comparators		Omadacycline	Comparators
Noel, et al. 2012	July 2007 and January 2008	11 sites in USA	≥ 18 years and cSSSI	118	116		100 mg q24 h	linezolid 600 mg iv q12h.
O’Riordan, et al. 2019 (OASIS-1)	June 2015 and May 2016	55 sites in 14 countries	≥ 18 years and ABSSSI	323	322		100 mg iv q12h/100 mg iv q24h or 300 mg po q24h	linezolid 600 mg iv/po q12h
Stets, et al. 2019	November 2015 and February 2017	86 sites in 26 countries	≥ 18 years and CABP	386	388		100 mg iv q12h/100 mg iv q24h or 300 mg po q24h	moxifloxacin 400 mg iv/po q24h
O’Riordan, et al. 2019 (OASIS-2)	Aug 2016and June 2017	33 sites in USA	≥ 18 years and SSSI	368	367		450 mg po q24h and 300 mg po q24h	linezolid 600 mg po bid
NCT00865280	April 2009 and April 2010	USA	≥ 18 years and cSSSI	70	73		100 mg iv q24h and 300 mg po q24h.	linezolid 600 mg iv/po q12h plus moxifloxacin 400 mg q24h iv/po
NCT03425396	January 2018 and June 2019	USA	≥ 18 years and cystitis	171	54		300 mg po q12h/ q24h 450 mg po q12h/300 mg po q24h 450 mg po q12h/q24h 450 mg q12h	nitrofurantoin 100 mg po q12h
NCT03757234	November 2018 and July 2019	5 countries	18–65 years and acute pyelonephritis	127	74		200 mg iv q24h 200 mg/100 mg iv q24h 200 mg iv/300 mg po or 100 iv q24h 200 mg iv/450 mg po or 100 mg iv q24h	levofloxacin 750 mg po/iv

cSSSI: Complicated Skin and Skin Structure Infections; CABP: community acquired bacterial pneumonia; SSSI: skin or skin structure infections; ABSSSI: acute bcterial skin and skin-structure infections

### Efficacy

**Fig. 4:**
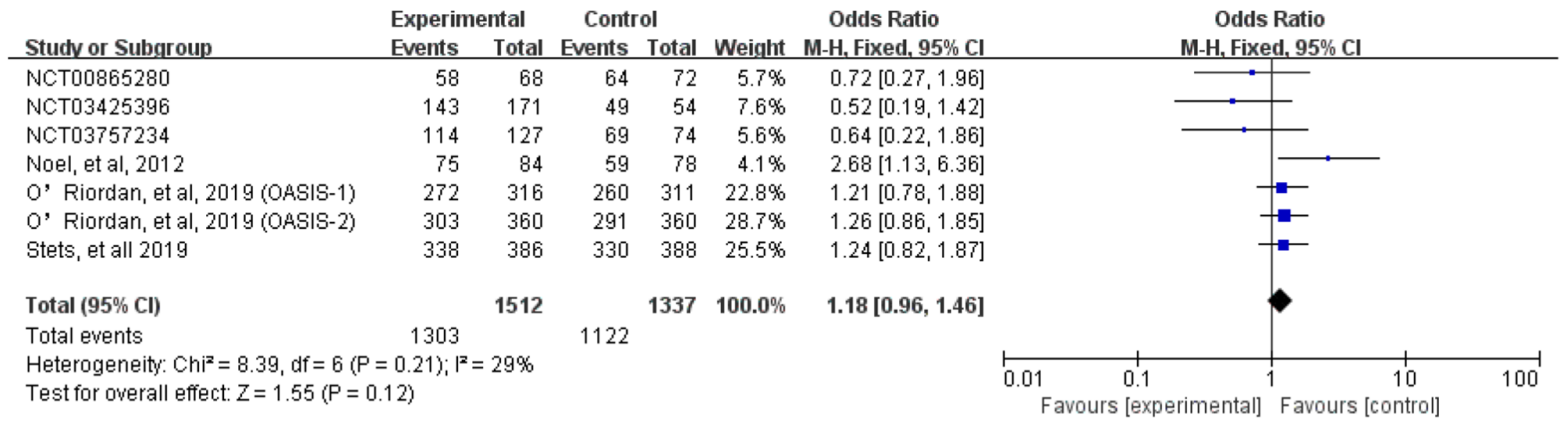
Overall clinical cure rates of omadacycline and comparators in the treatment of acute bacterial infections

**Fig. 5:**
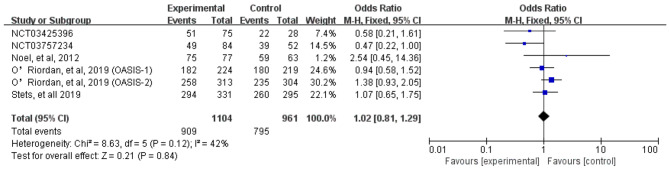
Overall microbiological eradication rates of omadacycline and comparators in the treatment of acute bacterial infections

**Fig. 6:**
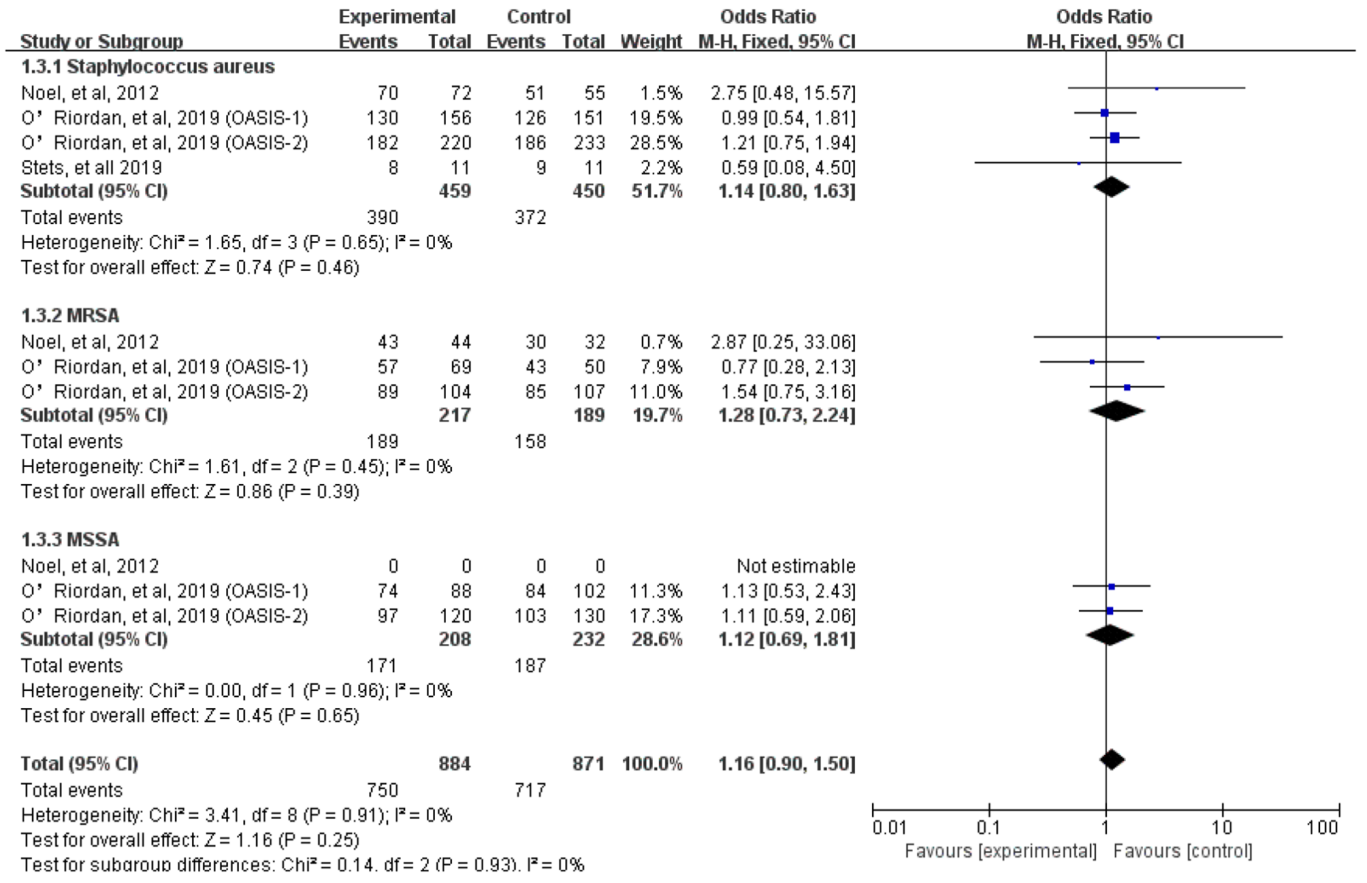
Overall *S. aureus* eradication rates of omadacycline and comparators in the treatment of acute bacterial infections

We assessed severity measures for efficacy, including clinical cure rates and microbiological eradication rates. Overall, our study illustrated that the clinical cure rates of omadacycline was similar to the comparators in the treatment of acute bacterial infections (OR = 1.18, 95% CI = 0.96, 1.46, I^2^ = 29%, Fig. 4) in the pooled analysis of 7 studies. In addition, omadacycline had a microbiological eradication rates similar to that of comparators in the treatment of acute bacterial infections (OR = 1.02, 95% CI = 0.81, 1.29, I^2^ = 42%, Fig. 5) in the pooled analysis. Four studies reported objective response rates among microbiologically evaluated populations; no statistical differences were observed between omadacycline and the comparators in terms of infection caused by *S. aureus* (OR = 1.14, 95% CI = 0.80, 1.63, I^2^ = 0%), MRSA (OR = 1.28, 95% CI = 0.73, 2.24, I^2^ = 0%), MSSA (OR = 1.12, 95% CI = 0.69, 1.81, I^2^ = 0%), and *E. faecalis* (OR = 2.47, 95% CI = 0.36, 16.97, I^2^ = 7%, Fig. 6).

Moreover, for the skin infection disease including complicated skin and skin structure infections, skin or skin structure infections, and acute bacterial skin and skin-structure infections, the clinical cure rates (OR = 1.29, 95% CI = 0.99, 1.68, I^2^ = 28%) and microbiological eradication rates (OR = 1.21, 95% CI = 0.90, 1.63, I^2^ = 8%) of omadacycline is not inferior to that of comparators. The same situation has been found in the clinical cure rates of urinary tract infections (OR = 0.57, 95% CI = 0.27, 1.18, I^2^ = 0%). But the microbiological eradication rates of urinary tract infections has higher in omadacycline than comparators (OR = 0.50, 95% CI = 0.27, 0.93, I^2^ = 0%).

### Safety

**Fig. 7:**
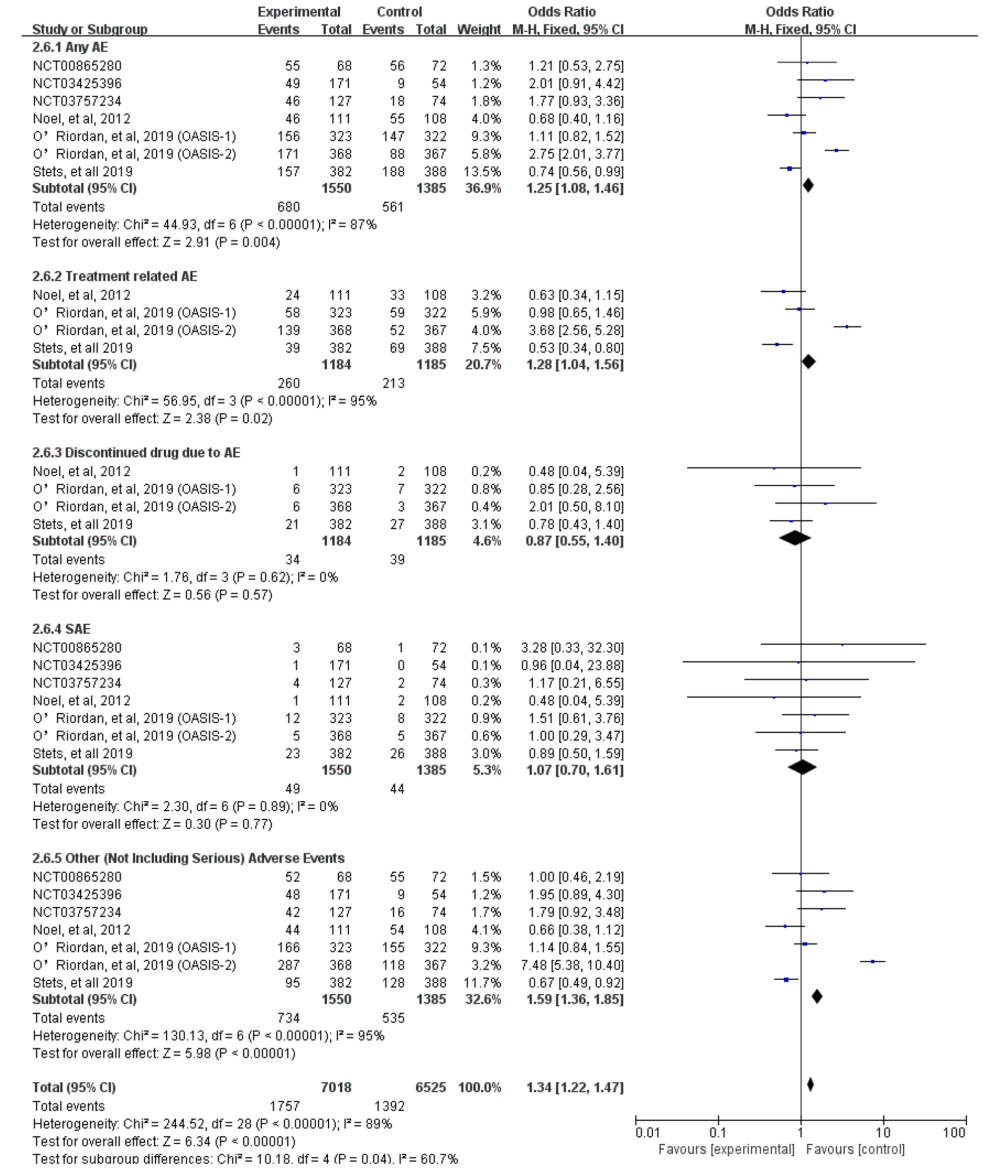
The risk of adverse events between omadacycline and comparators in the treatment of acute bacterial infections

We next assessed the incidence of AEs of treatment with omadacycline compared to other antibiotic treatments acute bacterial infection disease, including any AEs, treatment-related AEs, serious adverse events (SAEs), discontinuation of the study drug due to an AE, and the most common adverse events. A significant difference was found between omadacycline and the comparators for the risk of any AEs (OR = 1.25, 95% CI = 1.08, 1.46, I^2^ = 87%) and treatment-related AEs (OR = 1.28, 95% CI = 1.04, 1.56, I^2^ = 95%), respectively. In the sensitivity analysis, after removing the data from O’Riordan’s [19] OASIS-2 study, the heterogeneity of any AEs and treatment-related AEs decreased from 87 to 61% and 95 to 56%, respectively. Serious adverse events (SAEs, 3.2%) did not differ between the omadacycline and the comparators (OR = 1.07, 95% CI = 0.70, 1.61, I^2^ = 0%). Finally, the risk of discontinuation of the study drug due to an AE was lower for omadacycline than for the comparators (OR = 0.87, 95% CI = 0.55, 1.40, I^2^ = 0%) (Fig. 7). In the pooled analysis, all-cause mortality did not differ between the omadacycline and the comparators (OR = 1.40, 95% CI = 0.56, 3.49, I^2^ = 0%).

Gastrointestinal disorders were the most common AEs in this study, including vomiting, nausea, diarrhea, and constipation. Over all, the risk of gastrointestinal disorders was significantly increase in patients taking omadacycline than in those taking comparators (OR = 2.08, 95% CI = 1.73, 2.49, P < 0.00001, I^2^ = 95%). In the subgroup analysis of different types of gastrointestinal disorders, there was no significant difference between omadacycline and the comparators for the risk of constipation (OR = 2.08, 95% CI = 0.96, 4.48, P = 0.06, I^2^ = 0%), and a significant difference between omadacycline and the comparators for the risk of diarrhea (OR = 0.46, 95% CI = 0.31, 0.68, P < 0.0001, I^2^ = 66%), vomiting (OR = 2.09, 95% CI = 1.49, 2.94, P < 0.0001, I^2^ = 77%), and nausea (OR = 1.91, 95% CI = 1.50, 2.42, P < 0.00001, I^2^ = 85%). In the sensitivity analysis, after removing the data taken from the OASIS-2 study by O’Riordan [19], the heterogeneity of gastrointestinal adverse reactions, nausea, vomiting, and diarrhea—was decreased from 95 to 88% (P = 0.03), 85 to 47% (P = 0.52), 77 to 0% (P = 0.80), and 66 to 39% (P < 0.00001), respectively. In addition, there was no significant difference for the live function tests of aspartate aminotransferase increased (AST, OR = 0.83, 95% CI = 0.54, 1.29, P = 0.41) and alanine aminotransferase increased (ALT, OR = 0.87, 95% CI = 0.59, 1.30, P = 0.51) between omadacycline and the comparators.

## Discussion

Half a century has passed since the discovery of tetracycline [20]. They have been widely used in hospitals for treating infectious diseases. However, tetracycline-resistant isolates have been found in hospitals. Omadacycline is a third-generation tetracycline antibacterial agent for the treatment of CABP and ABSSSI [6, 7, 20]. Some results from in vitro studies indicated that omadacycline has broad antimicrobial activity against clinical pathogens. The in vitro study results indicated the effectiveness of omadacycline for ABSSSI, cystitis, acute pyelonephritis, and CABP in adult patients. A total of 168,519 clinical isolates were tested in seven in vitro studies [21–27]. The results show that omadacycline is active against G-negative, G-positive, and atypical pathogens, including MRSA and extended-spectrum β-lactamase-producing positive bacteria. However, it has also been used to treat other acute infections [28]. This meta-analysis based on seven RCTs found that the clinical cure rates and microbiological eradication (*S. aureus*, MRSA, MSSA, and *E. faecalis*) of omadacycline were not inferior to those of other comparators in the treatment of patients with acute bacterial infections. The findings are consistent with those of the Lan et al. study results, which show that the clinical efficacy of omadacycline is not inferior to that of comparators in the treatment of acute bacterial infections in adult patients [29]. However, in a Bayesian network meta-analysis by Li et al. [12], whose findings showed that omadacycline was associated with a higher rate of clinical and microbiological treatment success for the treatment of infection disease, our results contrasted that.

In terms of safety, the risk of AEs is another important concern. The pooled risks of any AEs and treatment-related AEs were higher for omadacycline than the comparators in this study. This is consistent with some studies from the past. The study by O’Riordan et al. [19] discovered AEs higher in omadacycline than linezolid (54% vs. 37%); and the risk of AEs was found to be higher in the omadacycline group than comparators in the studies by Stets et al. [18] (41.1% vs. 48.5%) and Noel et al. [16] (41.4% vs. 50.9%). And the study by Li et al. found that omadacycline was associated with a moderate rank of AEs, compared to another optional antimicrobial [12]. But contrary to the Lan et al. study [29] and the O’Riordan et al. study [17], the study by Lan et al. found no significant differences between omadacycline and comparators [29]; O’Riordan et al. [17] discovered a similar safety profile (48.3% vs. 45.7%). Importantly, the SAEs and all-cause mortality did not differ between the omadacycline and the comparators. Only three studies reported the deaths [17–19], and in total, 9 patient deaths were reported in the omadacycline group of two studies [17, 18], 3 deaths occurred in the linezolid group [17, 19], and 4 in the moxifloxacin group [18].

In addition, gastrointestinal disorders were the most frequent adverse events in this study. In this study, there was a significant difference between omadacycline and the comparators for the risk of gastrointestinal disorders (diarrhea, vomiting, and nausea), but no such difference for the risk of constipation. The findings are consistent with a study by O’Riordan et al. that showed mild to moderate nausea (30% vs. 8%) and vomiting (17% vs. 3%) in the omadacycline and linezolid groups [19]. In addition, another study also reported that the most common AEs were gastrointestinal (10.2% vs.18.0%), with the most significant difference being diarrhea (1.0% vs. 8.0%) [18]. Then, the gastrointestinal AEs were reported in 21 (18.9%) patients in the omadacycline group and 20 (18.5%) in the linezolid group, respectively [16]. Gastrointestinal disorders were reported as the most common adverse events in another new tetracycline derivative. In a meta-analysis reported, the risks of nausea (6.5%, 41/629) and vomiting (3.8%, 24/629) in the eravacycline group were higher than those in the comparator group, but these differences did not reach statistical significance (for nausea, RR = 4.79, 95% CI = 0.84–27.14, I^2^ = 70%; for vomiting, RR = 1.46, 95% CI = 0.76–2.81; I^2^ = 0%) [30]. When patients are treated with omadacycline, gastrointestinal disorders should be caution.

Among patients with a normal baseline ALT level, the change in ALT level was more than three times the upper limit level and was similar in the omadacycline and linezolid groups (1% vs. 4%) [19]. Levels of ALT or AST greater than 3 times the upper limit occurred in the omadacycline group (3.5% and 1.6%), in the moxifloxacin group (4.5% and 3.2%), respectively [18]. There was no significant difference in the live function tests of AST and ALT between omadacycline and the comparators. In total, approximately 3% of the included studies had elevated ALT and AST to varying degrees, indicating that we should be cautious with patient liver function during clinical use. Moreover, omadacycline is structurally similar to tetracycline-class of antibacterial drugs and may have similar adverse reactions, including abnormal liver function tests, hyperphosphatemia, and pancreatitis, etc. But discontinue therapy if any of these adverse reactions are suspected.

Therefore, the findings of this meta-analysis suggest that omadacycline is as safe as other comparators in the treatment of acute bacterial infections. In this study, 7 RCTs were considered in this meta-analysis, and 4 types of acute bacterial infections (complicated akin and skin structure infection, ABSSSI, CABP, cystitis, and acute pyelonephritis) were included. However, this study still has several limitations. First, all the included RCTs were funded by pharmaceutical companies. This might have caused the results to show good efficacy for the patients who received treatment with omadacycline in the real world. Second, some of the included trials were small samples. The results and conclusions should therefore be interpreted with caution. Finally, our study is limited to cases of suspected or confirmed G-positive pathogen infection. Future research should evaluate the efficacy and safety of these drugs in patients.

## Conclusion

In conclusion, omadacycline is as good as comparators in terms of efficacy and tolerance in the treatment of acute bacterial infections in adult patients. Thus, omadacycline is an appropriate option for antibiotic therapy in adult patients with acute bacterial infections. However, given the quality of the evidence, additional confirmation of the real-world study’s conclusion or larger sample size in RCTs are required.

## Data Availability

All the data supporting this systematic review are from previously reported studies and data sets, which have been cited. The processed data are available from the corresponding author upon request.
